# A Hijab-Effect Too? Clients’ Reflections on Professionalism and Empathy Toward Hijab-Wearing Public Servants

**DOI:** 10.1177/0734371X241234264

**Published:** 2024-03-04

**Authors:** Katharina Dinhof, Jurgen Willems, Noortje de Boer

**Affiliations:** 1 Vienna University of Economics and Business, Vienna, Austria; 2 Utrecht University, Utrecht, The Netherlands

**Keywords:** religious minority public servants, public sector employment, Hijab, religious stereotyping, public service encounters

## Abstract

Religious symbols, such as the hijab, are often deemed undesirable or banned in public employment. We test if clients’ perceptions and their performance are influenced by a hijab-wearing public servant, and further test if clients’ reflections on empathy or professionalism about the public servant mitigate potential negative effects. We preregistered and conducted a two-step 2 × 3 between-subjects experiment (*n* = 2,680; representative sample in Austria). We find no evidence that the wearing of a hijab by a public servant negatively influences clients’ perceptions, nor their performance during a public service process. The reflection answer with respect to professionalism or empathy, however, is related to clients’ performance: Clients’ positive reflection on public servants’ empathy or professionalism—independent of whether the public servant wears a hijab or not—positively relates to their performance in terms of task correctness. We discuss the relevance of these results regarding religious stereotyping and public employment policies.

## Introduction

The wearing of religious symbols in public employment, such as the hijab, is highly discussed in several countries around the world, specifically in Western-oriented countries with non-Muslim majority (e.g., Howard, 2017, 2021). This debate is due, in part, to the tension between demands for preserving the personal freedom of employees, while concurrently wanting to ensure bureaucratic neutrality (Lipsky, 2010; Weber, [1922] 1978). Religious symbols in the workplace raise concerns that these symbols displayed by public employees could negatively impact service interactions with clients.

When clients negatively stereotype public servants, and act upon these derogatory attitudes, this behavior can reduce the effectiveness of public services (e.g., Davidovitz & Cohen, 20212). Negative service encounters can thus result in higher frustration levels, but also in lower clients performance, such as the termination of a service by the client, or decreased client cooperation (e.g., Bitner et al., 1990; Bhattacharya & Sen, 2003; Guul, 2018; Habel et al., 2016; Varadarajan & Rajaratnam, 1986; Wieseke et al, 2012).

Drawing from the justification-suppression model of prejudice (Crandall & Eshleman, 2003), members of religious-minorities, specifically Muslims, have been found to suffer from negatively biased interactions (e.g., Ghumman & Ryan, 2013; Hebl et al., 2002, 2007; E. B. King & Ahmad, 2010). According to this theoretical model, people justify and express prejudice toward Hijab-wearing individuals, rather than suppressing them, because of negative stereotypic beliefs (e.g., fanatical, lazy, incompetent, and inefficient; Agerström & Rooth, 2009; Al-Sharif et al., 2018; Derous et al., 2017; Rooth, 2010).

Such negative prejudiced interactions are also evident in citizen-state interactions and may lead to less effective services (e.g., Assouline et al., 2022; Ghumman & Ryan, 2013; J. E. King et al., 2014; Pfaff et al., 2021; Wieseke et al., 2012). Within a public service context, we focus specifically on the performance of clients in encounters with frontline public servants. From the administrative burden literature, we know that public service usage is associated with costs imposed on the client (Burden et al., 2012; Herd & Moynihan, 2019). Compliance costs, for example, refer to all the efforts needed to meet administrative demands and procedures by the client such as filling out paperwork. In this study, we focus on filling out paperwork as a form of citizen performance during citizen-state encounters as it may clarify how cognitive processes at the side of clients—with respect to stereotyping and actual behavior in citizen state-interactions—influence public service outcomes. This perspective has been fairly neglected within existing literature (e.g., Döring, 2019). By documenting how clients’ expectations and practices are shaped by stereotypes and the expression of prejudice, public service encounters can be understood, and designed with greater consideration. Emerging interventions can therefore contribute to the reduction of biases and service failures, and ultimately lead to more pleasant interactions, and more efficient service outcomes.

Crandall and Eshleman’s (2003) justification-suppression model proposes that there are factors that suppress the negative effects of prejudice (in our case: toward hijab-wearing public employees). We theorize that two factors that could serve as suppressors are (1) experienced empathy toward the public employee by the citizen and (2) the perceived professionalism of the public employee (Pilling & Eroglu, 1994). While we do not propose that these are the sole factors that could suppress negative effects in citizen-state encounters, both are highly relevant in public sector contexts. Firstly, empathy has long been empirically recognized as a suppressor (Crandall & Eshleman, 2003). Moreover, there is growing empirical evidence that empathy and related emotions such as compassion play a pivotal role in citizens’ perceptions of—and behavior toward public employees (e.g., Jensen & Pedersen, 2017; Szydlowski et al., 2022). Second, unlike compassion, perceived professionalism has not been recognized as a suppressor. However, we argue there is evidence from literature on interactions in both public and private settings that it could function as such (e.g., Döring & Willems, 2023; Keh & Xie, 2009). We theorize that both factors hinder the expression of prejudice toward hijab-wearing public employees because empathy enables symbiotic interactions between employees and clients and perceived professionalism positively affects service satisfaction (e.g., Gabbott & Hogg, 2001; Varadarajan & Rajaratnam, 1986; Wieseke et al., 2012).

Against this background, we first examine (Research Question 1) *whether hijab-wearing public servants influence clients’ perceived professionalism and empathy toward them* and further ask (Research Question 2) *whether reflecting on professionalism or empathy toward the public servant moderates the effect of a hijab on clients’ performance outcomes in a public service.*

To answer these research questions, we conducted a pre-registered 2 × 3 between-subjects experiment in Austria (*n* = 2,680). The experiment took place in an online public service setting, where clients connect with a street-level public employee, who is represented by a portrait and an audio recording. Digital public service encounters are becoming increasingly common as more public organizations adapt digital technologies to improve service efficiency (Lindgren et al., 2019). In a two-step procedure, respondents were, in the first step, randomly assigned to the short audio instruction, about the upcoming performance-task (i.e., filling out forms correctly), explained by a public servant, with or without hijab. Based on the audio instruction, respondents rated the public servant’s professionalism, their empathy toward the public servant, or were assigned to no rating (passive control group). In the second step, these three reflection task groups were used as experimental interventions in step 2. After this reflection, respondents completed the instructed task for which their performance was measured. With this two-step procedure, we can thus first test if a hijab worn by a public servant influences perceived empathy and professionalism, as well as citizen performance in the public service process. In the second step, we can test if the (type of) reflection task has an additional moderating effect on citizen performance.

The experiment was carried out in Austria in the year 2021. In Austria, Muslims represent about 8% of the population (Austrian Integration Fund [ÖIF]), which makes Islam the largest minority religion in the country. Islam has been legally recognized as a religious community in Austria, giving Muslims the same rights as other legally recognized religious communities. Nevertheless, the social climate toward Muslims in Austria is tense and Muslims commonly experience discrimination (Heath & Martin, 2013; Wildan & Husein, 2021). The wearing of a headscarf and the spontaneous association of Islam regularly revive discussions around secularism (i.e., the separation of religion and state; Elver, 2014), which unintentionally gives rise to secular populist movements and systematic and interpersonal Islamophobia (Cesari, 2012; Hafez, 2017; Sezgin, 2019).

Employers from companies in European countries, including Austria, can generally rely on the ruling of the European Court of Justice (ECJ), which allows to restrict the display of religious symbols under certain conditions, such as the wearing of the Muslim headscarf (Howard, 2021; Salihović-Gušić, 2023). This ruling is based on the rationale of maintaining a neutral appearance to avoid associations with a particular religion or ideology. Thereby it exactly reflects the status of headscarf-bans in the Austrian public employment sector. To date, the headscarf-ban has been enforced in the Austrian police, among judges, and in the public rescue service. Other public employees, such as teachers or employees at a magistrate may continue to wear a headscarf at work (e.g., BBC, 2020). Moreover, many public services are outsourced to semi-public and/or nonprofit organizations, including hospitals, where headscarves are not banned in the forefront.

Moreover, access to public sector employment in Austria is limited for migrants due to bureaucracy, Austrian citizenship, or language (Organization for Economic Co-operation and Development Organization [OECD], 2014; Solano & Huddleston, 2020). Among these other barriers, the headscarf-ban might be one reason why minority-groups remain underrepresented in public employment, specifically in the police force, in education, and managerial positions (Jankowski et al., 2020; Lewis & Pitts, 2011; Nolan-Flecha, 2019; OECD, 2014).

Our research significantly contributes to the literature on religious stereotyping in public service encounters. First, while existing public administration literature on stereotyping is predominantly descriptive, we move this debate forward, by testing consequences of (religious) stereotyping (e.g., Bertram et al., 2022; de Boer, 2020; Dinhof et al., 2023; Willems, 2020). Second, the testing of how to counter stereotypes, or debias citizen-state interactions, remains rare (Nagtegaal et al., 2020). This study contributes thus to this agenda by testing interventions that can mitigate potential negative effects. Moreover, by drawing from the justification-suppression model of prejudice (Crandall & Eshleman, 2003), we introduce a suitable theoretical framework to understand the contributing factors of stereotyped citizen-state interactions. Concretely, by integrating the mitigating effects of empathy and perceived professionalism as potential suppression factors of prejudice, we verify and expand this model.

Practically, findings from this research offer useful directions for potentially more effective and less prejudiced public service encounters. From a policy perspective, this research offers a valuable empirical contribution to the global debate on hijab-bans in public employment by explicitly questioning current employment policies and the role of religious symbols in ensuring neutrality (Howard, 2017, 2021). From a human resource perspective, insights from this study might inspire recruiting and hiring practices for a more diverse public sector workforce and help prioritize employees freedom of expression and their well-being (Koch et al., 2015; Minghua, 2022; Yu, 2023).

## Stereotypes Toward Public Servants

Categorizing the social environment allows for quickly responding to situations or certain role occupants (Fiske, 2019; Judd & Park, 1993). When classifying individuals into certain social categories or groups, easily identifiable characteristics, such as gender, skin-color, but also religious attire or symbols, are used for this categorization process, and further elicit expectations (i.e., stereotypes; Friehs et al., 2022). Stereotypes are commonly shared beliefs or expectations about how members of a social group (should) behave (Fiske, 2019; Vescio & Weaver, 2013). To illustrate, an individual can be perceived as a member of a certain religious group, such as Muslims, because of physical attributes, symbols, or behaviors that others categorize as typically Muslim (Allport et al., 1954; Fiske, 2019).

While stereotypes refer to positive and negative beliefs about members of a social group, prejudice exclusively refers to the negative aspect of a stereotype, manifested in a concrete negative attitude or a negative feeling toward members of a social group. Accordingly, stereotype content substantially serves as an underlying justification for prejudice (Crandall & Eshleman, 2003). The public management and human resource management (HRM) literature addressing stereotypes and its consequences has grown over the past few years (e.g., de Boer, 2020; Harrits, 2019; Lotta & Kirschbaum, 2021; Pfaff, et al., 2021; Pedersen et al., 2018; Raaphorst et al., 2018; Willems, 2020). Recent research in public management and public administration has started to investigate how clients stereotype the public servants they meet (Bertram et al., 2022; de Boer, 2020; Döring & Willems, 2023; Willems, 2020). Associated stereotypes about public servants are either positive and contain associations such as helpful, prosocial, competent, and warm, or negative, such as risk-avoiding, lazy, and by implication slow and inefficient (de Boer, 2020; van de Walle, 2004; Willems, 2020). Such negative stereotypes manifest in negative attitudes or behaviors toward public servants, justify discriminatory behavior, and also harm effective public service outcomes for clients and employees (Davidovitz & Cohen, 2022; Del Pino et al., 2016; Porumbescu et al., 2021). Moreover, literature in human resource management link stereotypes to discriminatory practices, specifically in terms of recruiting processes, hiring practices and behaviors in the organization (e.g., Alteri, 2020; Riccucci & Saldivar, 2014; Rubin & Alteri, 2019; Yu, 2023). Overall, it has an impact on professional encounters in many ways, but especially on those who are stereotyped. Due to identifiable membership of particular social groups, stereotyping is automatic and highly prevalent, not least in public service encounters (e.g., Hansen, 2022; Harrits, 2019).

### The Effect of Public Servants Wearing Hijabs on Clients’ Evaluations

Due to the clear identifiability of characteristics related to religious affiliation, the wearing of a Muslim headscarf (e.g., hijab) as a visible religious symbol represents a salient cue for categorizing into social group membership (e.g., Allen & Nielsen, 2002; Bodenhausen & Peery, 2009; Unkelbach et al., 2010). In that sense, it has been argued that Muslim women are more likely than men to encounter discrimination because their attire visibly conveys their religious identity (Allen & Nielsen, 2002; Weichselbaumer, 2020). Empirical evidence indicates that the Muslim headscarf—precisely, hijabs for women and turbans for men—triggers a range of negative stereotypes toward Muslims and even manifests in aggressive behavior toward them (e.g., Pfaff et al., 2021; Unkelbach et al., 2008, 2009, 2010). Accordingly, Muslim men are more likely to be stereotyped as fanatical, radical, potentially violent, oppressing, and having (other similar) values and traits that are considered as incompatible with western norms, and in particular public service norms of secular states (e.g., Antepli, 2010; E. B. King & Ahmad, 2010; Mujtaba et al., 2016). Muslim women, on the other hand, are more likely stereotyped as submissive, abused, oppressed, unattractive, and less intelligent (e.g., Barkdull et al., 2011; Derous et al., 2012; Mahmud & Swami, 2010). These presumptions have been shown to lead to decreased empathy toward religious minority groups in informal, but also professional interactions (e.g., Balliet et al., 2014; FitzGerald et al., 2019; Ghumman & Jackson, 2010; E. B. King & Ahmad, 2010; J. E. King et al., 2014).

Moreover, such negative stereotypes imply that Muslim men and women pose a challenge to workplace integration, professionalism, and organizational performance. For one thing, they are ascribed different work styles, because their religious expression of the Islamic faith is seen as intensive in practice, which does not always align with typical Western work cultures (Mujtaba et al., 2016; Schuster & Weichselbaumer, 2022; Zaheer, 2007). Second, studies have shown that Muslims are more likely to be associated with behavioral traits of incompetence, unprofessionalism, inefficiency, laziness and slowness, and lacking initiative (Agerström & Rooth, 2009; Al-Sharif et al., 2018; Derous et al., 2009; Derous et al., 2017; Rooth, 2010). This set of negative beliefs leads to unfavorable stigmatization of Muslim employees that ultimately results in workplace discrimination (e.g., Al-Sharif et al., 2018; Ghumman & Ryan, 2013).

For example, research points to a selection bias in hiring practices in the sense that women who wear a Muslim hijab are rejected more quickly compared to applicants who do not wear a hijab (Unkelbach et al., 2010). However, other research on employability provides slightly different findings: In this regard, Ghumman and Jackson (2010) found that hijab-wearing women, compared to others with different religious identifiers, were rated most employable. Similarly, no differences appeared in the offers for job interviews for job applicants who wore Muslim attire, compared to others (E. B. King & Ahmad, 2010). However, negative attitudes toward Muslims might be more prevalent in interpersonal and less overt discriminatory behavior (e.g., Hebl et al., 2002, 2007). Accordingly, interactions with applicants identified as Muslim were found to be shorter and more negative (E. B. King & Ahmad, 2010). Similarly, a Muslim co-worker was rated lower on competence, less desirable in terms of a working relationship, and was perceived to make an organization less attractive in the eyes of highly religious Christian colleagues. No negative effects were found for colleagues who signaled that they were less religious (J. E. King et al., 2014).

Concerning public employment, similar negative effects are present in citizen-state interactions. Accordingly, evidence suggests that Muslims are facing discrimination by public servants due to their religion in the form of lower response rates from school principals (Pfaff et al., 2021). Similarly, it is found that Jewish doctors reject Muslim clients more than Jewish clients (Assouline et al., 2022). Even though these studies first documented effects of religious symbols in the public sector—the focus has solely been on how public servants negatively discriminate toward religious-identifiable clients. However, we argue that Muslim public servants who wear a hijab, are equally prone to the discrimination by clients. Clients can show this on the one hand through devaluation and negative interactions, which can lead to the termination of the service and ultimately result in less efficient service performance (e.g., Assouline et al., 2022; Ghumman & Ryan, 2013; J. E. King et al., 2014; Pfaff et al., 2021; Wieseke et al., 2012).

On the other hand, when clients hold prejudice toward so-called outgroup public servants (i.e., public servants who are not part of the same social category of clients), the unfamiliarity with an outgroup, as well as situational uncertainty, can cause stronger affective responses, most notably anxiety, anger, and feelings of discomfort (e.g., Schmader et al., 2015). This well-documented intergroup anxiety can lead to performance losses by impacting clients’ cognitive capacity (e.g., Trawalter et al., 2009). Specifically, regulating affective responses during encounters occupies emotional and cognitive resources. While it is noteworthy to mention that concrete performance-related findings in this context are scarce, there is evidence that prejudiced inter-group interactions (mainly regarding ethnic- or racial-groups), result in higher cognitive load and decreased cognitive flexibility, which can further lead to performance losses (e.g., Mendes & Koslov, 2013; Stephan, 2014).

Accordingly, since prejudice and/or negative stereotypes might be involved when individuals from different social categories (hijab- vs. non-hijab wearing individuals) interact, the proposed service encounter may be explained using the justification-suppression model of prejudice (Crandall & Eshleman, 2003). The theoretical model suggests that prejudice, defined as a negative attitude about a group, can result in expressions of discrimination when suppression is reduced and justification occurs, for example by stereotypes. Suppression is understood to be “an externally or internally motivated attempt to reduce the expression or awareness of prejudice” (Crandall & Eshleman, 2003, p. 420). More specifically, the justification-suppression model of prejudice proposes that “genuine” prejudice toward certain groups is not expressed because of suppression. Suppression factors include, for instance, norms and values, accountability, discomfort, or feelings of empathy (Shapiro & Neuberg, 2008).

In contrast, prejudice *is* expressed when justifications occur. More precisely, prejudice describes “a negative evaluation of a social group or a negative evaluation of an individual that is significantly based on the individual’s group membership” (Crandall & Eshleman, 2003, p. 414). Justification therefore refers to “any psychological or social process that can serve as an opportunity to express prejudice without suffering external or internal sanction” (Crandall & Eshleman, 2003, p. 425). The justification-suppression model of prejudice explicitly classifies stereotypes about certain groups as an example of such a justification (Crandall & Eshleman, 2003). Thus, negative stereotypical ascriptions toward hijab wearing Muslims may result in the justification of discriminatory behavior toward them. In the case of public service interactions, clients may hold negative stereotypes toward hijab-wearing public servants and thus justify discriminatory evaluations of professionalism and decrease their empathy toward them. We therefore hypothesize the following:

*Hypothesis 1a*: A hijab-wearing public servant is responded to with less empathic feelings by clients, compared to a public servant who does not wear a hijab.
*Hypothesis 1b*: A hijab-wearing public servant is perceived as less professional, compared to a public servant who does not wear a hijab.
*Hypothesis 1c*: A hijab-wearing public servant causes lower citizen performance in the public service process, compared to a public servant who does not wear a hijab.

### The Effects of Reflecting About Empathy or Professionalism on Performance

To adapt the theory of justification-suppression of prejudice (Crandall & Eshleman, 2003) to the public service context, we use service literature to incorporate suppression factors that (1) are relevant and implementable in service interactions, while at the same time (2) serve to mitigate negative effects of stereotyping to conquer justification of prejudice.

Accordingly, high levels of empathy and perceived professionalism are two important indicators of successful professional interactions, particularly in the service context between clients and employees (Jensen & Pedersen, 2017; Pilling & Eroglu, 1994). Since service encounters typically take place in a dyad, an effective service interaction depends, among others, on the level of empathy apparent in the interaction (Gabbott & Hogg, 2001; Goodsell, 1981). Within a service context, empathy has often been referred to as “the ability to relate to others” (Ranchordas, 2021, p. 1341), and described as an important component of social cognition that enables humans to understand and respond adaptively to others’ emotions (Gabbott & Hogg, 2001; Spreng et al., 2009). Important from a practical management perspective is, that empathy represents a skill that can be developed and improved through practice and is thus valuable to be researched in (public) service settings (Edlins, 2021; Edlins & Dolamore, 2018; Jensen & Pedersen, 2017; Wieseke et al., 2012). For this research, we rely upon these definitions, and we conceptualize empathy as “the ability to acknowledge, respond, and understand the situation of others” (Ranchordas, 2021, p. 1367).

Evidently, empathy is associated with a variety of positive interactional characteristics: it enables clients’ perspective-taking, exerts feelings of compassion, and enhances clients’ sensitiveness and caring for employees (e.g., Beatty et al. 1996; Berry et al., 2002; Bitner et al., 1990; Jensen & Pedersen, 2017).

At the same time, the act of reflecting on someone else’s state might lead to a reduction in uncertainty. This can happen through gaining a better understanding of the other person, as well as through role assurance provided by the execution of a specific task (e.g., Avery et al., 2009).

Similarly, by enabling a more accurate understanding of the interaction partner, empathy also leads to symbiotic interactions between employees and clients (Håkansson & Montgomery, 2003; Wieseke et al., 2012). This results in improved client satisfaction and loyalty, a higher willingness to cooperate, more forgivingness toward the employee, as well as increased trust and expectations related to future interactions with the employee (Aggarwal et al., 2005; Gabbott & Hogg, 2001; Wieseke et al., 2012). If empathy is absent, interactions are perceived as less enjoyable and are less effective in terms of performance for the client and the employee (e.g., Wieseke et al., 2012).

Moreover, empathy has been shown to be decreased in religiously biased interactions (e.g., Assouline et al., 2022; Balliet et al., 2014; FitzGerald et al., 2019; Ghumman & Jackson, 2010; E. B. King & Ahmad, 2010; J. E. King et al., 2014; Unkelbach et al., 2010; Pfaff et al., 2021). Particularly toward religious minority groups, it has also been found to serve as a factor to mitigate and suppress discriminatory behavior due to negative stereotypes (e.g., Crandall & Eshleman, 2003; E. B. King & Ahmad, 2010). Since empathic feelings include the ability to understand the situation of others (Ranchordas, 2021), we argue empathy toward others can be evoked by thinking carefully and deeply, in other words *reflecting*, about a person’s state of being (e.g., Ochsner et al., 2004). Reflecting on empathic feelings might thus ultimately serve as a suitable intervention (Vogel & Willems, 2020), suppressing the turn from stereotypes to prejudice.

Against this background, we suggest that (1) reflecting on empathy toward a service employee (i.e., a public servant), as well as (2) the actual reflection score, which refers to a high or low experienced empathy toward the employee, enable clients to perform better in a public service process:

*Hypothesis 2a*: An active reflection task on empathy toward the public servant mitigates the negative effect of a hijab-wearing public servant on clients’ performance in the public service process (compared to no reflection).
*Hypothesis 3a*: The reflection score given as a result of a reflection task on experienced empathy mitigates the negative effect of a hijab-wearing public servant on clients’ performance in the public service process.

While empathy has empirically been recognized as a suppressor within the justification-suppression-model (Crandall & Eshleman, 2003), we expand the theory by hypothesizing and testing perceived professionalism as another relevant suppression factor in service interactions. Indeed, perceived professionalism of an employee has been found to affect interactions between employees and clients. The professional image of an employee reflects how to behave appropriately in a given occupational context and is argued to be an essential characteristic in service provisions (e.g., Evetts, 2003; Döring & Willems, 2023; Uhlmann et al., 2013). Similarly, professionalism relates to an individual’s ability to meet normative expectations by effectively accomplishing job-related tasks, toward the professional community (e.g., colleagues) and clients (Roberts, 2005).

In that sense, a professional reputation positively affects clients’ service satisfaction (e.g., Varadarajan & Rajaratnam, 1986), and has a positive influence on customer trust and identification (e.g., Keh & Xie, 2009), as well as on customer loyalty (e.g., Nguyen & Leblanc, 2001; Srivastava & Sharma, 2013). Similarly, Pilling and Eroglu (1994) find high levels of perceived professionalism positively influence employees’ sales performance and thereby conclude that professionalism substantially contributes to successful service interactions. Furthermore, the reflection task regarding professionalism may also be helpful because it sharpens the role of the other person as a public servant, and one’s own, as a client. This could lead to a higher level of role assurance in the interaction and reduce uncertainty. The reduction of situational uncertainty seems to play a role in intergroup anxiety, which might lead to performance losses (Schmader et al., 2015; Trawalter et al., 2009).

However, research points to negative stereotypes toward Muslims, labeling them as less intelligent, incompetent, inefficient, lazy, slow, and who lack initiative in the workplace (e.g., Agerström & Rooth, 2009; Al-Sharif et al., 2018; Derous et al., 2009, 2012, 2017; Rooth, 2010). These stereotypes per se contradict ascriptions of professionalism to Muslim employees, which leads to potentially less favorable and less effective service encounters. In this regard, negative service encounters that are marked by discriminatory behavior due to religious stereotyping, might result in, for example, higher frustration levels, the termination of a service, or decreased client motivation, effort, or cooperation (e.g., Assouline et al., 2022; Guul, 2018; E. B. King & Ahmad, 2010; J. E. King et al., 2014). Hence, a client’s performance in a public service process might seriously decrease if service interactions involve stereotyping. Thus, we again apply the process of *reflecting* about experienced professionalism to mitigate the negative effects of stereotyping on clients’ performance in the public service process. Precisely, we suggest that (1) a reflection task on a public servant’s professionalism, as well as (2) the reflection score on perceived professionalism, which refers to high or low experienced professionalism, enable clients to perform in the public service process more effectively:

*Hypothesis 2b*: An active reflection task on a public servant’s professionalism mitigates the negative effect of a hijab-wearing public servant on clients’ performance in the public service process (compared to no reflection).
*Hypothesis 3b*: The reflection score given as a result of a reflection task on perceived professionalism mitigates the negative effect of a hijab-wearing public servant on clients’ performance in the public service process.

## Method

The research protocol for this study was pre-registered via AsPredicted (see https://aspredicted.org/blind.php?x=MCY_MOD). Data is available at Open Science Framework.

### Design and Procedure

**Figure A1.**
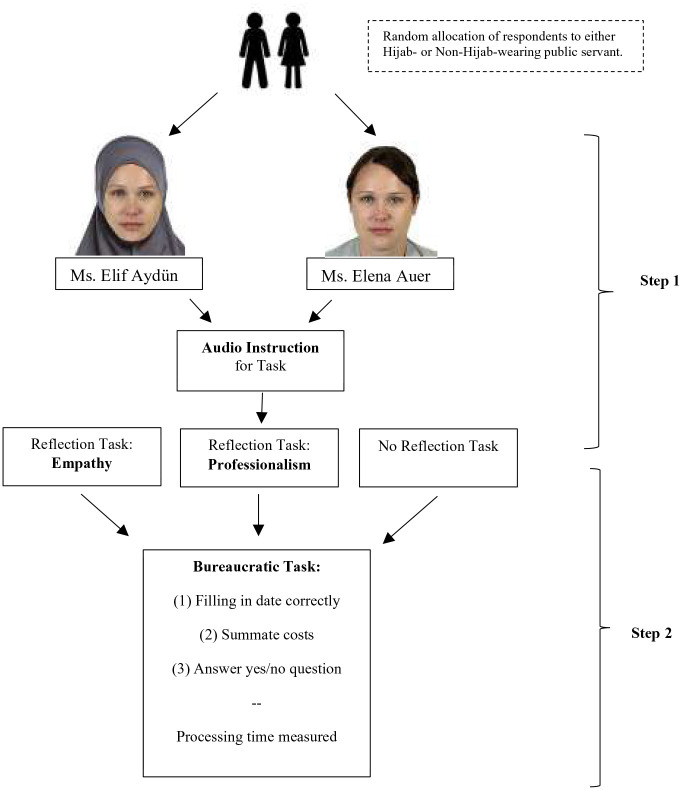
Two-step experimental procedure.

We conducted a two-step 2 (public servant with vs. without hijab) × 3 (reflection task: professionalism vs. empathy vs. no reflection) between-subjects experiment, with two dependent variables in the first step and two dependent variables in the second step. A visualization of the experimental procedure can be found in Figure A1 in the Appendix.

#### Step 1

In the first step, respondents were randomly assigned to a vignette, presenting a photo of a public servant with or without hijab, along with a short introduction of the public servant as either “Ms. Elif Aydün” or “Ms. Elena Auer” (detailed information can be found in the Appendix). The wearing of a hijab, as well as the name of the public servant were varied, resulting in a typical Muslim name (Elif Aydün) for the hijab-wearing public servant versus a non-Muslim name (Elena Auer) for the public servant without hijab. This manipulation should increase the salience of the hijab as a Muslim identity cue, adding to external validity (e.g., Derous et al., 2017).

The actual face of the female public servant—which was the same in both vignettes—was taken from the Chicago Face Database version 2.0.3 (Ma et al., 2015), while the hijab was added for one version of the picture. Facial expressions were validated elsewhere to be affect-neutral (see Ma et al., 2015). Figure A1 (Appendix) depicts the portraits of the public servants.

While seeing the photo with the short introduction, respondents were asked to listen to a two-minute audio recording from that public servant. The audio recording included detailed instructions for processing a bureaucratic task. We chose for the combination of a picture, a short introduction with first- and last name, as well as an audio recording (rather than an instruction video by a person), because then we could manipulate only the aspect of a hijab and the name, while the verbal instruction is identical. The audio recording was spoken by a native German-speaking woman.

After listening to the instructions, respondents were randomly assigned to one of three groups. In the first group (Group 1), respondents were asked how empathetic they felt toward the public servant. For respondents in this group, we test Hypothesis 1a based on the difference of the first treatment. In the second group (Group 2), respondents were asked to report how professional they perceived the public servant. For this group, we test Hypothesis 1b based on the difference of the first treatment. In the third group, no reflection tasks were asked, and thus, this group represents the control group for step 2. Hypothesis 1c is tested for all groups, in an integrative analysis (see further, as well as in the preregistration).

The two items for measuring empathy toward the public servant were “I can easily imagine the employee’s daily work routine,” and “I can put myself in the employee’s shoes and imagine how she feels in her daily work routine,” while the two items for measuring perceived professionalism were “The employee conveyed the instructions in a professional manner,” and “The employee’s instructions were clear and understandable.” Items were adapted from established measures by Edlins and Dolamore (2018) and McCluney et al. (2021). For the items, respondents indicated their extent of agreement on a 7-point Likert-scale, ranging from −3 (*strongly disagree*) to +3 (*strongly agree*). Item responses for the professionalism, as well as for the empathy measure, were averaged to form a scale. The Cronbach’s alpha for the empathy-scale is .88 and .85 for the professionalism-scale.

#### Step 2

**Figure A2.**
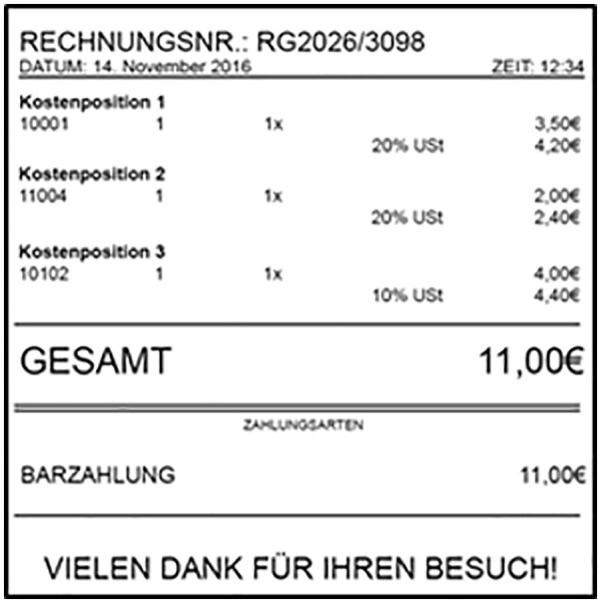
The invoice respondents saw to solve the bureaucratic task. Depicted in German language.

In the second step, the questions about empathy and perceived professionalism also served as reflection tasks themselves. After respondents were assigned randomly in either the empathy, professionalism, or passive treatment, they were asked to complete the task for which they were receiving instructions in Step 1. The task required processing information regarding an invoice (for example in the case of a tax declaration). An image of the invoice can be found in the Appendix (as Figure A2). In relation to the instruction, respondents were asked (1) to fill in the date in a correct format, (2) to summate cost elements, and (3) to answer a yes/no-question based on the type of invoice (the exact wording of the questions can be found in the Appendix). From this, a “correctness index” was built. The “correctness index” is operationalized as a score between 0 and 3, depending on the number of correct answers given. Additionally, processing time for the task was measured in seconds.

The correctness index and the processing time are two operationalizations of performance, which are both estimated as the dependent variables for testing Hypothesis 1c and for testing the mitigation effects in Hypotheses 2a and 2b, as well as 3a and 3b. The comparison of Group 1 (empathy reflection) versus the passive control group tests Hypothesis 2a, and the comparison of Group 2 (professionalism reflection) with this passive control group tests Hypothesis 2b. By adding the actual value answered in the respective reflection tasks, we can test Hypotheses 3a and 3b. Moreover, to ensure data quality, two attention checks were included. The first one recorded whether respondents have spent at least two minutes on the survey page where the instruction of the public servant was presented (which took approximately 2 minutes to listen to). The second one checked whether respondents correctly recognized the stimuli materials by asking whether the woman in the picture was wearing a headscarf (hijab), with responding options of “yes,” “no,” or “I don’t know.” Finally, demographics were asked.

### Respondents

The sample size has been determined by an ex-ante power analysis. Accordingly, in order to discover a small effect size (*f* = 0.10), at least 215 (214.7) respondents are necessary in each treatment group. Hence, a sample of at least 1,290 participants is suitable to uncover a small effect size in a 6-group comparison, which is based on a power analysis for ANOVA (2 × 3 full factorial design), using the package “pwr” in R (Champely, 2018). Parameters for the aforementioned power analysis are: number of groups (*k*) = 6, power = 0.80, significance level = 0.05, and the effect size is set to *f* = 0.10 (small effect).

Questions were formulated in German. Relying on the professional panel provider Qualtrics, a representative Austrian sample (representative regarding gender, age, and region (federal states in Austria) consisting of 3,506 respondents was collected. The sample for the main analysis was reduced to the 2,680 respondents who met the pre-registered attention checks.

The sample for analysis is gender-balanced (49.50% female, 0.06% diverse), with a mean age of 40.64 years (*SDage* = 15.81). With respect to education, 5.69% finished primary school as the highest level of education, 40.26% finished vocational school, 33.57% finished high school, and 20.46% attended a university. The majority of respondents have an Austrian nationality (71.11%), and 10.81% have a first-generation migration background, while 7.92% have a second or third-generation migration background. Regarding religious affiliation, 52.99% identify as Christians (Catholic, Orthodox, Protestant), 4.93% identify as Muslims, while 39.45% do not profess any religion or indicate to belong to other religions (2.62%).

## Analysis and Results

Descriptive statistics regarding perceived professionalism of the public servant, empathy toward the public servant, and performance in terms of correctness and processing time are presented in Table 1.

**Table 1.** Descriptive Statistics of Perceived Professionalism, Experienced Empathy, Task Correctness, and Processing Times.

Descriptive statistics of dependent variables				
***Step 1***: *Professionalism and empathy evaluations as dependent variables*				
			Hijab	No hijab
Perceived professionalism (Mean^a^)		1.99	0.92	
Experienced empathy toward the public servant (Mean^a^)		1.91	0.85	
***Step 2***: *Performance measures as dependent variables*		Empathy reflection	Professionalism reflection	No reflection
Scores of correctness index^b^	Hijab	2.15	2.16	2.22
	No hijab	2.26	2.23	2.27
Processing time in seconds (mean)	Hijab	95.78	81.56	86.63
	No hijab	88.39	94.24	84.56

a Respondents indicated their extent of agreement on a 7-point scale, ranging from −3 (*strongly disagree*) to +3 (*strongly agree*).

b“ Correctness index” is operationalized as a score between 0 and 3, depending on the number of correct answers given in three tasks.

### Hypotheses 1a to 1c: Hijab-Effects on Perceived Professionalism, Empathy Toward the Public Servant, and Clients’ Performance

For step one, two-tailed *t*-tests were conducted to examine the mean differences in perceived empathy (H1a) and professionalism (H1b) toward a public servant with and without hijab. Moreover, the hijab-effect is also tested on respondents’ performance (H1c) in the group that did not receive a reflection task. Performance is operationalized by the correctness index and the processing time of the instructed bureaucratic task. To test whether the wearing of a hijab by the public servant influenced clients’ performance, two OLS regressions were conducted, separately for the correctness index and the processing times of the task.

#### Findings Regarding Hypotheses 1a and 1b

Regarding empathy toward the public servant: The hijab-wearing public servant (*M* = 0.919) is responded to with equal empathy as the public servant who does not wear a hijab, *M* = 0.850; *t*(886.43) = −0.704, *p* = .481. Similar results are found for perceived professionalism: Results reveal that the hijab-wearing public servant (*M* = 1.99) is perceived as equally professional as the public servant who does not wear a hijab, *M* = 1.90; *t*(908.51) = −0.951, *p* = .342. Thus, Hypotheses 1a and 1b are not supported.

#### Findings Regarding Hypothesis 1c

Tables 2 and 3 report the results of the OLS regressions in which we predict citizen performance based on the experimental treatments, as well as the reflection scores. Findings point to no differences regarding clients’ task correctness when the task was instructed by a public servant with hijab, compared to a public servant without hijab (Model 1: β = −0.05, *p* = .488, Table 2). Moreover, regarding clients’ processing times, no differences are found when clients processed a task instructed by a public servant with hijab, compared to a public servant with no hijab (Model 1: β = 2.07, *p* = .858, Table 3). In conclusion, hijab-wearing public servants do not cause lower citizen performance, compared to public servants who do not wear a hijab. Hence, Hypothesis 1c is also not supported.

**Table 2.** OLS Regression Explaining Response Correctness on Bureaucratic Task.

Dependent variable: Correctness									
	Model 1			Model 2			Model 3		
Predictors	Estimates	CI	*p*	Estimates	CI	*p*	Estimates	CI	*p*
(Intercept)	2.27	[2.17, 2.36]	<.001	2.27	[2.17, 2.36]	<.001	2.27	[2.17, 2.36]	<.001
Hijab [with hijab]	−0.05	[−0.18, 0.08]	.488	−0.05	[−0.17, 0.08]	.472	−0.05	[−0.17, 0.08]	.472
Reflection task [professionalism]	−0.04	[−0.17, 0.10]	.595	−0.46	[−0.60, −0.32]	<.001	−0.51	[−0.66, −0.35]	<.001
Reflection task [empathy]	−0.01	[−0.14, 0.13]	.912	−0.20	[−0.33, −0.06]	.004	−0.22	[−0.35 to −0.08]	.002
Hijab [with hijab] × Reflection [professionalism]	−0.03	[−0.21, 0.16]	.771	−0.05	[−0.22, 0.13]	.608	0.05	[−0.17, 0.26]	.678
Hijab [with hijab] × Reflection [empathy]	−0.05	[−0.24, 0.13]	.568	−0.07	[−0.25, 0.11]	.448	−0.03	[−0.21, 0.16]	.773
Reflection score				0.22	[0.19, 0.25]	<.001	0.25	[0.20, 0.29]	<.001
Hijab [with hijab] × Reflection score							−0.05	[−0.11, 0.02]	.138
Observations	2,680			2,680			2,680		
*R*^2^/*R*^2^ adjusted	.002/.000			.070/.068			.071/.068		

**Table 3.** OLS Regression Explaining Processing Time of Bureaucratic Task.

Dependent variable: Processing time									
Predictors	Model 1			Model 2			Model 3		
	Estimates	CI	*p*	Estimates	CI	*p*	Estimates	CI	*p*
(Intercept)	84.56	[68.08, 101.04]	<.001	84.56	[68.08, 101.03]	<.001	84.56	[68.09, 101.03]	**<.001**
Hijab [with hijab]	2.07	[−20.54, 24.67]	.858	2.07	[−20.53, 24.67]	.858	2.07	[−20.53, 24.66]	.858
Reflection task [professionalism]	9.68	[−13.12, 32.48]	.405	3.17	[−21.98, 28.32]	.805	12.15	[−15.39, 39.69]	.387
Reflection task [empathy]	3.83	[−19.31, 26.97]	.745	0.93	[−22.68–24.54]	.939	4.93	[−19.20, 29.06]	.689
Hijab [with hijab] × Reflection [professionalism]	−14.75	[−46.39, 16.89]	.361	−15.04	[−46.68, 16.59]	.351	−32.39	[−70.75, 5.97]	.098
Hijab [with hijab] × Reflection [empathy]	5.32	[−26.50, 37.15]	.743	5.09	[−26.73, 36.91]	.754	−2.77	[−36.07, 30.52]	.870
Reflection score				3.42	[−2.15, 8.98]	.229	−1.30	[−9.40, 6.81]	.754
Hijab [with hijab] × reflection score							8.91	[−2.23, 20.06]	.117
Observations	2,680			2,680			2,680		
*R*^2^/*R*^2^ adjusted	.001/−.001			.001/−.001			.002/−.000		

### Hypotheses 2 and 3: The Effects of Reflecting About Empathy or Professionalism on Clients’ Performance

The OLS regressions in Tables 2 and 3 also test Hypotheses 2a and 2b, that is, if a reflection task about public servant’s empathy or professionalism, influences the negative relationship between “wearing a hijab” and clients’ task performance (Table 2 for the correctness, Table 3 for the processing time).

Moreover, to test Hypotheses 3a and b, additional OLS regressions were conducted (Tables 2 and 3, Models 2 and 3), where the actual empathy or professionalism rating is taken into account in addition to the mere reflection in itself.

In Models 2 and 3 (Table 2 for the correctness, Table 3 for the processing times), the additional variable “Reflection Score” is added. This variable is coded with value 0 if a person was not given a reflection task and is coded with the respondent’s value given on the reflection score between −3 and + 3 for the respective reflection task that the respondent received. Given the fact that the reflection task was answered on a scale with 0 as the neutral middle option and given the model specification in Tables 2 and 3, this variable can now be interpreted in the same way as an interaction term between reflection task type and score given for that reflection. This means that the intercepts in Models 1 and 2 indicate the average correctness scores of a citizen who did not see a hijab-wearing public servant, and who did not receive the reflection opportunity (both modeled as the reference categories; the average correctness score of the reference categories is 2.27 (*p* < .001), the average time is 84.56 (*p* < .001). For a respondent who received the professional reflection task, the dummy variable for that treatment (in all three models) gives the effect of the reflection task in itself (regardless of the score given on that reflection task). These dummies test if reflection tasks have an effect, and the interaction with the hijab treatment tests Hypotheses 2a and 2b.

The additional variable “Reflection Score” in Models 2 and 3, in both Tables 2 and 3, gives thus the additional effect of the actual score when a reflection task was answered, and has to be interpreted in combination with the dummy coded reflection task categories. The main effect of this variable tests if the content of the reflection task has a direct effect on clients’ performance. Concretely, it tests whether clients perform better when they are more empathic toward the public servant or when they perceive the public servant as more professional. The interaction of this variable with the hijab treatment thus tests Hypotheses 3a and 3b.

#### Findings Regarding Hypotheses 2a and 2b

For *correctness* (Table 2), results in Models 1 indicate no significant main effects for the empathy reflection task (M1: β = −0.01, *p* = .912), nor for the professionalism reflection task (M1: β = −0.04, *p* = .595). Similarly, no significant effects for both reflection tasks are found for *processing times* (M1, empathy: β = 3.83, *p* = .745; professionalism: β = 9.68, *p* = .405; Table 3). Importantly, no significant interaction effects of the professionalism or empathy reflection task with the hijab-treatment are found for *correctness* (M1, professionalism: β = −0.03, *p* = .771; empathy: β = −0.05, *p* = .568; Table 2), nor *processing times* (M1, professionalism: β = −14.75, *p* = .361; empathy: β = 5.32, *p* = .743; Table 3). As a result, *Hypotheses 2a and 2b* on clients’ performance *are not supported.*

However, by adding the respondents’ scores of the reflection questions on empathy or professionalism to the model (Model 2), findings indicate that the scores of respondents on the reflection tasks positively influence respondents’ performance regarding correctness (M2: β = 0.22, *p* < .001, Table 2). We do not find this effect for processing time of the task (M2: β = 3.42, *p* = .229, Table 3). That means that clients’ reflection on public servants’ empathy or professionalism—independent of whether the public servant wears a hijab or not—positively relates to their performance in terms of correctness. It is noteworthy, however, that by adding the “Reflection Score” measure (based on Models 2 and 3, compared to Models 1), the main effects of the reflection task dummies turn negative. This means that a reflection task in itself, that is rated by a respondent as neutral (i.e., the middle option out of seven options for the reflection task), has a negative effect in itself. Only if a respondent is substantially positive about the public servant (answering a +2 or +3), this negative effect is sufficiently countered.

#### Findings Regarding Hypotheses 3a and 3b

Model 3 adds the extra interaction of ‘Reflection Score’ with the hijab treatment. This effect is not significant for *correctness* (M3: β = −0.05, *p* = .138, Table 2), nor for *processing times* (M3: β = 8.91, *p* = .117, Table 3). *Hypotheses 3a and 3b* are thus *not supported.*

Given the nature of the dependent variable (values for the correctness index ranging from 0 to 3, in ordered form, but potentially not continuous), the same analysis with a probit ordered model was conducted for robustness reasons. Findings do not differ as compared to findings depicted in Table 3.

#### Complementary Analysis for Testing Correctness Separately for Tasks

In a final complementary analysis (also pre-registered) we tested these models with a logistic regression of the separate tasks regarding correctness. This means that each task was coded as correct (1) or incorrect (0) and for each of these variables, the three models were predicted with a logistic regression. The findings about the effects of reflection tasks on performance (correctness) are confirmed for each of the separate tasks. Hence, we can claim that this is a finding that is consistent across different tasks. We can thereby rule out that the hypotheses would be supported for one task only (or part of a task), but was swamped in the integrated analysis. This means that none of the hypotheses are supported for the separate tasks.

## Discussion

By drawing from literature on religious stereotypes in public service encounters, this research investigated two assumptions: First, we investigated whether hijab-wearing public servants negatively influence clients’ perceptions on professionalism and experienced empathy toward them. Second, we examined whether reflecting on professionalism or empathy toward the public servant, in turn, serve as suppression factors that mitigate negative hijab-effects on clients’ performance in a public service process (Crandall & Eshleman, 2003). While we find that the reflection scores regarding professionalism or empathy evaluations of the public servant positively relate to clients’ performance for task correctness, we find no evidence that the wearing of a hijab by a public servant negatively influences clients’ perceptions, nor their performance during a public service process. However, the reflection scores on professionalism or empathy, which are a result of the reflection tasks, positively relate to clients’ performance regarding task correctness: A positive evaluation of the public servant increases clients’ task correctness. This effect, though, is independent of whether the public servant wears a hijab. We reach these conclusions after examining effects on three conceptually different variables—experienced empathy, professionalism, and citizen performance—tested in a well-powered (*n* = 2,680), pre-registered, and carefully designed experiment.

These findings could indicate that transferring this effect into a *public service context* is less straightforward. Indeed, while a considerable body of research finds formal and particularly interpersonal discrimination against Muslims, a smaller collection of research does not find negative effects of a hijab, or of religious stereotyping toward Muslims (e.g., Ghumman & Ryan, 2013). Our study—which had a specific public sector focus—also cannot confirm this effect. From this perspective, we postulate that the public sector context might have special conditions that can affect the occurrence of a hijab-effect.

First, we argue that public service provision might be perceived as inherently secular and clients expect that public servants’ religion (i.e., Islam) should and does not play a role in service provision. Precisely, client’s common knowledge and expectations on how secularism is applied—for example with respect to how public servants are trained and how service provision is executed—might ironically serve as a buffer for the emergence of religious bias. Hence, personal religious symbols *of employees* might play no or a smaller role for clients, and religious stereotypes might have less possibility of occurrence. However, this alternative interpretation needs further verification in different sectoral contexts. By testing hijab-effects separately for private versus public services, along with varying perceptions of secularism, causal evidence regarding moderating effects can be drawn.

Additionally, it could be theorized that while stereotypes were indeed activated during the encounter (Harrits, 2019), these might not have been applied toward the public servant. Concretely, potential stereotypes of this social group were not used for evaluating the public servant. There can be several reasons for this. While stereotypes are often considered to be activated automatically, research suggests that these activations can be controlled to prevent them from transferring to the application phase (Gilbert & Hixon, 1991; Rivers et al., 2020). In order to regulate and control for stereotypes, motivations and mental abilities play a role in a given situation (Devine, 1989; Fazio & Dunton, 1997).

Given that clients typically aim for a satisfactory outcome when using a public service, it is plausible that they actively manage or suppress stereotypes, refraining from applying them toward public servants.

Moreover, reflection questions of empathy and professionalism might have induced this reflection process that prevents activated stereotypes related to hijab-wearing from being used uncontrollably in an evaluation and its further impact on performance (e.g., Rees et al., 2019). Further research could investigate the conditions under which stereotypes are applied, or precisely where these are helpful and necessary and where these present a hindrance to effective public service.

In a similar vein, a public service interaction, other than an interpersonal social interaction, might already provide the interaction partners with a higher level of *certainty.* This certainty of a controlled and regulated public service could on the one hand lead to less reliance on (negative) stereotypes and to less inter-group anxiety with social out-group members (e.g., Avery et al., 2009). Interventions regarding professionalism and empathy might additionally reinforce situational certainty, as both reflection tasks provide heightened role assurance, which enables clients to navigate through the public service encounters.

However, role certainty, on the other hand, might also lead to certain relationship expectations. Accordingly, the public service relationship between employee and client is characterized by asymmetry. Clients strongly depend on a positive public service outcome (e.g., social aid, bureaucratic assistance). Therefore, evaluating a public service employee obviously negative, solely due to illegitimate personal preferences, could harm the client because the public servant might be less empathic or responsive to the needs of the client.

Finally, individuals’ motivation to control prejudice, due to societal pressures, might lead to compensatory behaviors toward minority-status group members (i.e., overly positive behavior; Bergsieker et al., 2010; LaCosse & Plant, 2020; Mendes & Koslov, 2013). In our experiment, this can manifest in evaluating the hijab-wearing public servant more favorably, compared to the public servant without hijab. Indeed, without explicit justification to openly express prejudice, the hijab effect could manifest in overcorrection, and not, as assumed, into negative discriminatory effects.

### Limitations

Our study has some methodological limitations. The first limitation refers to respondents socially desirable response-behavior. Due to self-presentation concerns, respondents might underreport socially undesirable attitudes, which complicates the documentation of (negative) attitudes and behavior toward hijab wearing individuals. However, although we cannot completely rule out socially desirable behavior, this experiment used anonymization in the answer process, assured confidentiality, and heightened the importance of telling the truth for the scientific character of the experiment, for reducing such measurement errors as best as possible (Krumpal, 2013). A second limitation refers to the experimental method. More specifically, our experimental scenarios do not fully reflect the complexity of the real world, and real-world interactions may be more likely to reveal prejudiced behavior. Thus, other data collection methods, such as qualitative interviews or focus groups or observations, should be considered to gain a deeper insight into real-world public service experiences. Regardless, as online public service provision has become more common over the years (Lindgren et al., 2019), the design of our study is—at least to some extent—comparable to real-world online service interactions. In addition, it is noteworthy to mention that our experimental manipulation included not only the hijab, but also the variation of the name, resulting in a typical Muslim name for the hijab-wearing public servant versus a non-Muslim name for the public servant without hijab. This additional manipulation increases external validity by highlighting the salience of the hijab as a Muslim identity cue (Bodenhausen & Peery, 2009; Derous et al., 2017). While increased salience could amplify the effect of the hijab, no effects are found. The last limitation concerns our measurement. Other than in the variables that we chose, a negative hijab-effect might also be detectable though other measurements, such as the willingness to cooperate with the public servant, or in nonverbal behaviors (e.g., facial expressions, eye contact, sweating, shorter interactions, less questioning) that reflect individuals’ genuine feelings and affective states (Avery et al., 2009; Blascovich & Tomaka, 1996; King & Ahmad, 2010). Hence, future research should observe the occurrence of prejudice in more natural settings, without restricting prejudice to the measurement of certain behaviors or evaluations (Assouline et al., 2022).

Our study also has theoretical limitations. First, we have applied Crandall and Eshlerman’s (2003) theoretical model to a particular type of stereotype: that of a female hijab-wearing public employee. Future research could further investigate stereotypes based on other personal and socio-demographic characteristics (e.g., gender, disability), and importantly their intersection (Dinhof & Willems, 2023). For example, ethnic or race-related stereotypes can be studied intersectional with gender-stereotypes, exploring whether men, wearing a turban or other religious attire, would yield to different effects. Second, our study solely concerns how the service receiver (in this case: the client) responds based on stereotypes that are prompted. We did not theorize beforehand any interaction effects based on individual-level characteristics such as ethnicity. Based on the literature on representative bureaucracy (e.g. Guul, 2018) it could be that a particular client-type, namely those that identify with the religious symbols portrayed by the public servant, responds- and performs differently that the client that does not identify with those same religious symbols. We did not test this micro-level effect of representative bureaucracy cues since this was not the core interest of this study. Future research could use sub-group analyses based on proxies for religious symbols such as religion at the individual-level because this could reveal if our null effects differ across specific sub-groups of clients.

## Conclusion

Against the claim that a decent public service performance or quality cannot be guaranteed when a public servant wears a hijab, we find *no* causal evidence that hijab-wearing public servants are affecting the performance-outcomes of clients (Lipsky, 2010; Weber, [1922] 1978). While wearing a hijab is generally found to be a source of religious or ethnic discrimination, these negative effects might not occur in the same way when public servants wear a hijab, at least not regarding clients’ public service performance. As such, these findings are important for both the theoretical and policy-related debates on religious symbols worn by public servants.

Theoretically, this research is among the first that introduces the hijab-effect, originally documented mainly regarding labor market and hiring discrimination (e.g., Mujtaba et al., 2016), to the public service setting. Yet, while heated debates are being held about secularism, Islam, and the headscarf ban (Sezgin, 2019; Weichselbaumer, 2020), little research is taking place in public administration contexts regarding religious stereotypes and minority public servants. Our research contributes to this gap and sheds light on how hijab-wearing public servants are perceived and reacted to from clients.

In doing so, this research also builds on the growing research agenda of public servant stereotypes. So far, the documentation of public servant stereotypes has been steadily growing in the public management literature (Bertram et al., 2022; de Boer, 2020; Willems, 2020). By experimentally studying effects of frequently stereotyped symbols (e.g., the hijab), we move beyond the predominantly descriptive findings on occupation-related stereotypes toward public servants. Moreover, these approaches do not (yet) differentiate between groups of public servants based on other stereotypical characteristics. Therefore, we also explicitly contribute to the vast research on religious stereotyping and discrimination in the public service. Although religious symbols are increasingly researched in this context (e.g., Assouline et al., 2022; Pfaff et al., 2021), available studies have primarily focused on the treatment of clients in relation to employees negative religious- or ethnic-related stereotypes. In contrast, the present research offers a novel perspective by investigating clients’ role as stereotyping agents toward public service employees. As such, insights of this research are also transferable to human resource literature that specifically addresses how employees with hiring responsibilities evaluate and treat minority job-seekers (e.g., Derous et al., 2009; Lippens et al., 2023; Zschirnt & Ruedin, 2016). It can be emphasized that how hijab-wearing applicants or employees are perceived is not only influential for hiring decisions (e.g., Allen & Nielsen, 2002; Weichselbaumer, 2020), but might also be influential on performance-oriented interaction outcomes with clients or employees. Moreover, this research also expands the previous research by investigating hijab-effects on the variable client performance. This is important because even though clients’ performance is considered one of the most important outcomes of public service provision (Davidovitz & Cohen, 2022), and commonly discussed in hijab-ban debates, it is not commonly researched as a dependent variable within inter-group service encounters.

Second, while we do not find effects of negative religious stereotyping or prejudice, the findings about impressions of perceived empathy and professionalism show that beliefs about others (i.e., stereotypes) *do* play a role in public service encounters. Drawing from the justification-suppression model of prejudice (Crandall & Eshleman, 2003), we offer a suitable theoretical framework to understand the contributing factors of stereotyped citizen-state interactions. Accordingly, negative stereotypical beliefs, which decrease perceptions of empathy and professionalism, might serve as a justification for prejudice. If additional justifications appear in a public service interaction, negative prejudice may well be acted out by clients. For example, an unpleasant service experience, caused by the delivery of a negative message from the public servant (e.g., the financial assistance is less than the client anticipated), might be perceived as a legitimate justification to express prejudice (Lynn et al., 2008).

Moreover, as the justification-suppression model of prejudice (Crandall & Eshleman, 2003) also allows to study the mitigating effects of empathy and perceived professionalism during prejudiced service encounters, we not only adapt this theoretical model to the (public) service context and verify it, but also extend it by perceived professionalism as another suppression factor to prevent prejudice. Professionalism, other than empathy, has not been researched in this regard, and yet not acknowledged as a suppression factor regarding the expression of prejudice (Uhlmann et al., 2013). However, this characteristic is increasingly addressed in the research of public sector stereotypes (Dinhof & Willems, 2023).

The third theoretical contribution refers to the scarce literature on countering stereotypes and debiasing strategies in citizen-state interactions (e.g., Nagtegaal et al., 2020). In our research, reflection tasks are used as a way of debiasing encounters susceptible to stereotyping. We find that the reflection scores on professionalism and empathy, resulting from the reflection tasks, positively influence clients’ performance and in turn increase service efficiency: If perceptions toward the public servant are positive, clients perform better.

Practically, several implications can be drawn from these findings for public service encounters and public employment policies. First, the findings are particularly useful for designing public services. Precisely, the findings that reflection questions can be used to evoke positive perceptions of empathy and work-related characteristics (e.g., professionalism) is very beneficial for the design of low-threshold interventions in service encounters. This can be realized, for example, with stimuli that refer to elements of the workplace or service-desk design (e.g., Emerson & Murphy, 2014): by placing stickers, posters or information sheets that are visible to the clients and encourage reflection about how they see and approach employees. Similarly, clients could be stimulated to develop a more individuated view of the employees, which could increase empathy and reduce the reliance on stereotypes (Singletary & Hebl, 2009). This could be realized by employees actively sharing or signaling unique information about themselves to a point where they feel comfortable—for example by simply introducing themselves by their full names. Additionally, service scripts or certain phrases (“I understand your frustration,” “I am very sorry to hear that”) can be used by employees to enhance mutual empathy, or to evoke an even more professional perception (Avery et al., 2009). Importantly, our findings also reveal that reflection questions only positively contribute to the service process when the impression will be favorable. In case of less favorable impressions, reflecting toward an employee might be even counterproductive, harming public service. Our results thus offer critical directives on how public service provision and debiasing strategies can be implemented (Ofir et al., 2009). Moreover, findings of how minority employees (such as hijab-wearing public servants) are perceived by the public is also essential for informing human resource practices that allow a more equal access to jobs and fair treatment at the workplace. Firstly, as it provides insights into processes (e.g., negative stereotyping, prejudice, cognitive load) that may impact workplace environment, the findings might inspire organizations to develop inclusive policies and training programs. For instance, by equipping hiring personnel with the knowledge to navigate diversity-related issues, organizations can enhance the fairness of their recruitment processes and minimize the impact of stereotypes on hiring decisions (e.g., Koch et al., 2015; Lee et al., 2015; Stephens et al., 2020). Moreover, organizations could implement concrete anti-discriminatory concepts in their mission to create and signal effort toward a climate of diversity among the employees. This might be particularly valuable for employees who choose to express their religious or cultural identity, such as through wearing the hijab. An inclusive environment might positively impact employees’ satisfaction and performance-oriented outcomes (e.g., Minghua, 2022).

Moreover, investigating and discussing how the public perceives minority employees, concretely hijab-wearing public servants, might shift the attention to challenges these minority employees face in the workforce. Insights from this research might thus be valuable for crafting communication and engagement strategies that promote tolerance among the public, but also increase interest for potential applicants. Human resource strategies could therefore proactively educate the public, fostering respectful encounters with their employees, and increase minority representation with effective recruiting for minority applicants (Linos, 2018).

Ultimately, we would like to highlight that organizations should actively seek to protect their employees from biases and discrimination through several measures (Roberson et al., 2003). On that note, the most important contribution of this research is the questioning of current employment policies and whether a hijab-ban in public sector employment is harmful or beneficial, and precisely for whom. Our results initiate a first valuable empirical contribution to the global debates on potential hijab-bans and challenge the present perspective that hijabs harm objective public service encounters. While secularism should ensure neutrality and the potential reduction of biases, it is also contrary to principle of equal treatment and further leads to the exclusion of certain social groups. In this debate, personal religious symbols or religious attire are seen as a threat to secularism (e.g., Rasheed & Garcia, 2020; I. U. Syed, 2021). In that sense, secularism can also be seen as a discriminatory and Western-oriented ideology that mainly benefits Western religious majorities (Salaymeh & Lavi, 2021). On the other hand, however, it is noteworthy to mention that concrete concerns of other social groups (e.g., based on religious affiliation) were not considered in this context. While our research documents perceptions of empathy and professionalism, it does not examine the concerns raised by social groups that have conflicting relations. This poses challenges for the public service.

Nonetheless, it can be argued that public organizations should create internal conditions that enable a high degree of objectivity and neutrality of employees when interacting with clients or filing decisions (e.g., through trainings or certain selection processes of applicants). Employees’ visible (religious) symbols should play less of a role in ensuring neutrality. Importantly, conditions of objectivity, neutrality and fairness should then also be signaled to the public.

Moreover, we would like to acknowledge the far-reaching negative consequences of inter-personal, and formal discrimination for minority group-members, especially for the Muslim population. Muslims are subject to greater penalties compared to other ethnic and religious minorities, and experience double discrimination because of their status as ethnic minorities, but also because of their religious affiliation (Heath & Martin, 2013). Importantly, as hijab-wearing women are predominantly affected by the public sector’s hijab-ban, they are systematically excluded from the workforce. By this action, the public sector not only deters suitable candidates by underutilizing human capital and decreasing employment attractiveness for a diverse pool of applicants, but it also fails the demands for a representative bureaucracy by reproducing discriminatory practices (e.g., Howard, 2021; Kingsley, 1944; Sievert et al., 2022).

In conclusion, our research advances the research agenda of religious stereotyping in the public sector. Given the ongoing debates about hijab-bans in public employment, we see this research as an important empirical contribution, and a necessary starting point, to elaborate on the effects of religious symbols in public employment. Considering the serious consequences for the Muslim population, it is precisely in the public service where this research agenda is likely to be of great importance.

## Appendix

### Descriptions and Experimental Stimulus Material

#### Introduction of the Public Servant

Respondents first read the following introduction of the employee (in German; translated to English) and were then exposed to the image of either a public servant with hijab, or without hijab.

Ms. Elena Auer is an employee of a municipal department of the City of Vienna. Among other things, she is responsible for customer service and develops innovative solutions to support applicants in the best possible way. She is also responsible for audio instructions (see below). Ms. Elif Aydün is an employee of a municipal department of the City of Vienna. Among other things, she is responsible for customer service and develops innovative solutions to provide optimal support to applicants. She is also responsible for audio instructions (see below).

#### Instruction for Listening the Audio Instruction for the Bureaucratic Task

After being exposed to the picture of the public servant with or without hijab, respondents read the following instruction.

Please listen to her instructions that will help you complete a task later in this survey. To listen to the instructions, please click on the play button below. You will need these audio instructions later on when filling out an invoice.

#### Description of Bureaucratic Tasks for Measuring Performance

After listening to the short audio instruction, respondents saw an invoice (Find an image of the invoice in Figure A2 in the Appendix text), for which they were answering the four questions (“Now please fill in the following three items/questions correctly in relation to the invoice (based on the instructions).”; “Please enter the invoice date in the correct format (according to the instructions):”; “Please total the individual NET cost items on the invoice and enter the total amount here:”; “Was the invoice issued by a private law or public law organization? (Recognizable in the invoice number.”^1^). Based on these questions, performance in form of correctness and processing time was measured. Respondents were able to see the stimulus material the while time while filling out these questions.

## Notes

1. Response options for question no. four were: public-law (0) or private-law (1).
